# Correction: A stretchable and biomimetic polyurethane membrane for lung alveolar in vitro modelling

**DOI:** 10.1038/s41598-026-37895-z

**Published:** 2026-02-16

**Authors:** Emmanouela Mitta, Andrew Gilmore, Angeliki Malliri, Sarah Harriet Cartmell

**Affiliations:** 1 Rothwell Building, Booth Street East, Manchester, M13 9PL UK; 2 Henry Royce Institute, The University of Manchester, Manchester, M13 9PL UK; 3 Division of Cancer Sciences, The University of Manchester, Manchester, M13 9PL UK; 4 Manchester Cell Matrix Centre, Oxford Road, Manchester, M13 9PT UK

Correction to: *Scientific Reports* 10.1038/s41598-025-98500-3, published online 25 April 2025

The original version of this Article contained an error.

As a result of an error during figure assembly, in Figure 4, the image for Day 4/TPU/100k cells was a duplication of the image for Day 4/TPU/200k cells. The incorrect Figure 4 along with its caption is provided below.

**Figure 4:**
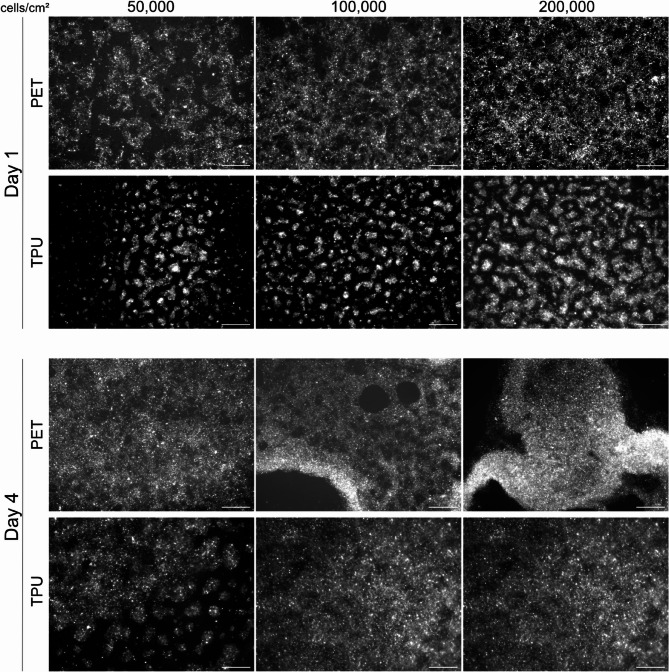
Representative fluorescent images of H441 cells seeded at different seeding densities on PET and TPU membranes on day 0 comparing day 1 to day 4 in culture. Cells were stained with DiI prior to seeding on either substrate.1

The original Article has been corrected.

