# Supplementary material for: A stretchable and biomimetic polyurethane membrane for lung alveolar in vitro modelling
Source: Sci Rep. 2025 Apr 25;15:14585. doi: 10.1038/s41598-025-98500-3 (PMC12032040; doi:10.1038/s41598-025-98500-3)
Supplement: Supplementary file 1 — Supplementary Information. [file 41598_2025_98500_MOESM1_ESM.docx]

**
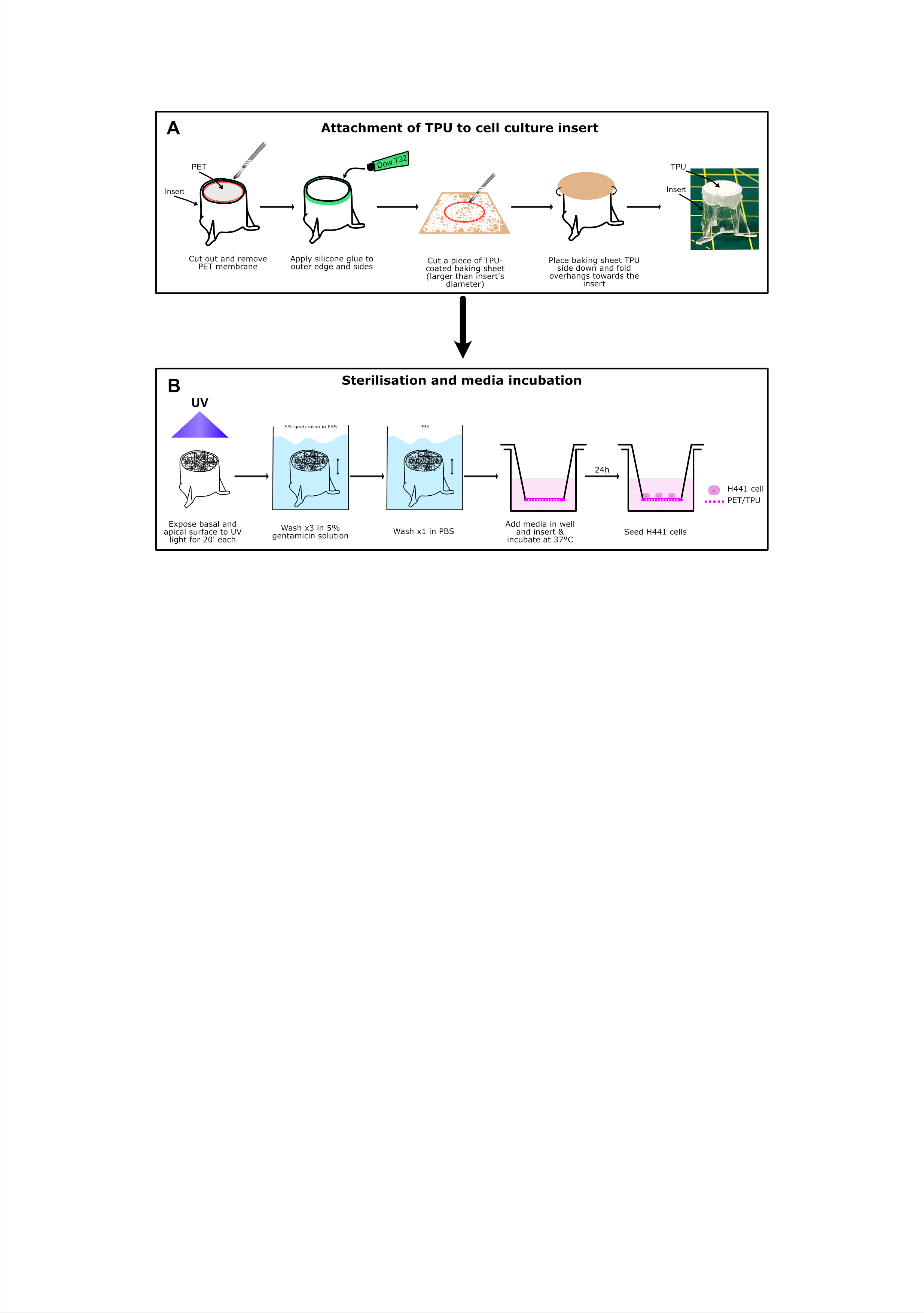
Supplementary Material**

**Figure S1 (A)** Process of attachment of electrospun TPU on cell culture inserts. Supplied PET membrane is removed with a scalpel and silicone adhesive is applied on the outer edges of the insert. A suitable round piece of TPU-coated baking sheet is cut out and placed TPU side down on top of the insert. Overhanging sides are folded down towards the insert. Carefully the baking sheet is removed leaving the TPU only attached to the insert. **(B)** Process of TPU sterilisation once on the insert. TPU inserts are exposed to UV light for 20 minutes either side (apical and basal). Then they are transferred and submerged to a 5% gentamicin solution three times to wash. Gentamicin is washed off in PBS once and inserts are transferred to appropriate plates and incubated with media for 24h. Once incubation is complete, scaffolds are seeded with the H441 cell line at appropriate seeding densities.


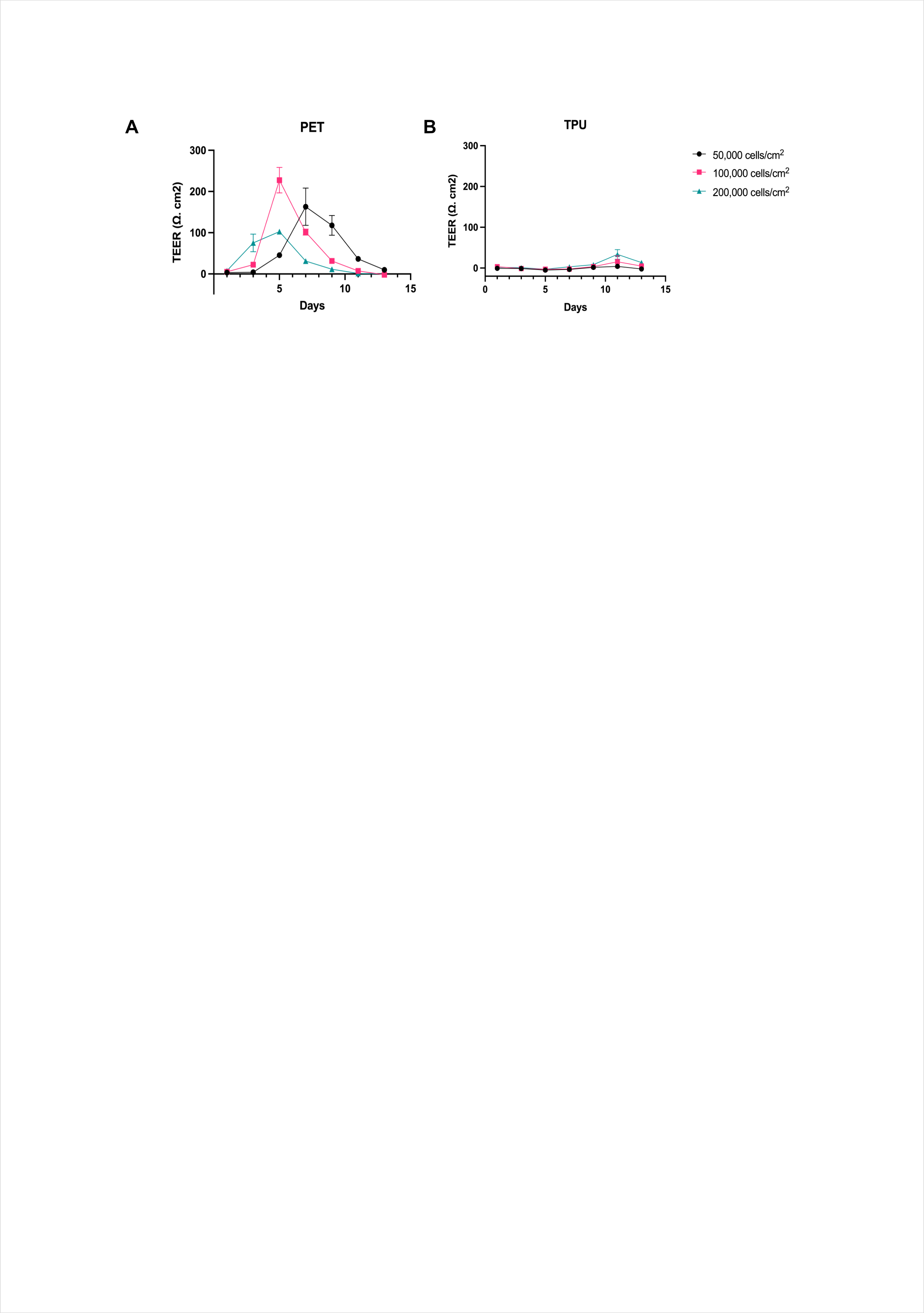


**Figure S2 (A-B)** Monitoring of barrier integrity of H441 cells seeded at different seeding densities on **(A)** PET, or **(B)** TPU scaffolds monitored over a period of 13 days (Mean ± SD). Scale bars: 500 μm.
